# Structural attributes for the recognition of weak and anomalous regions in coiled-coils of myosins and other motor proteins

**DOI:** 10.1186/1756-0500-5-530

**Published:** 2012-09-25

**Authors:** Margaret S Sunitha, Anu G Nair, Amol Charya, Kamalakar Jadhav, Sami Mukhopadhyay, Ramanathan Sowdhamini

**Affiliations:** 1 National Centre for Biological Sciences (TIFR), UAS-GKVK Campus, Bellary Road, Bangalore, 560 065, India; 2 VLife Sciences Technologies Private Limited, Aundh, Pune, 411 007, India

**Keywords:** Coiled-coil, Charged-patch, Hydro ladder, Pseudoenergies, Charged clusters, Heptads, Knobs-into-holes packing

## Abstract

**Background:**

Coiled-coils are found in different proteins like transcription factors, myosin tail domain, tropomyosin, leucine zippers and kinesins. Analysis of various structures containing coiled-coils has revealed the importance of electrostatic and hydrophobic interactions. In such domains, regions of different strength of interactions need to be identified since they could be biologically relevant.

**Findings:**

We have updated our coiled-coil validation webserver, now called COILCHECK+, where new features were added to efficiently identify the strength of interaction at the interface region and measure the density of charged residues and hydrophobic residues. We have examined charged residues and hydrophobic ladders, using a new algorithm called CHAHO, which is incorporated within COILCHECK + server. CHAHO permits the identification of spatial charged residue patches and the continuity of hydrophobic ladder which stabilizes and destabilizes the coiled-coil structure.

**Conclusions:**

The availability of such computational tools should be useful to understand the importance of spatial clustering of charged residues and the continuity of hydrophobic residues at the interface region of coiled-coil dimers. COILCHECK + is a structure based tool to validate coiled-coil stability; it can be accessed at http://caps.ncbs.res.in/coilcheckplus.

## Background

Structure and function of proteins are primarily determined from their amino acid sequences. The alpha-helical coiled-coils are simple structural units that consist of repeating blocks of seven residues which are commonly termed as 'heptads'. They are mostly seen as protein-protein interaction domains which mediate many vital functions in the system like oligomerization, cell division, transport of macromolecules, mobility and transcription. Coiled-coils consist of two or more helices wound around as a superhelix - like strand of a rope. On the basis of the number of helices involved in coil formation, they are differentiated into higher order structures like dimers, trimers, tetramers and pentamers [1-4]. The most commonly observed coiled-coil types are dimers with two α-helices and trimers with three α-helices wrapped around each other into a left-handed superhelix.

Coiled-coils usually contain a repeated pattern, “HPPHCPC” of hydrophobic (H), polar (P) and charged (C) amino acid residues referred to as heptad repeat. The position of the heptad repeat is usually labeled as '*abcdefg*', where ‘a’ and ‘d’ positions are ideally occupied by the hydrophobic residues and ‘e’ and ‘g’ are occupied by oppositely charged residues. The crucial fact in the structure of coiled-coils is the burial of the hydrophobic residues to form a core region, permitting it to form an amphipathic structure, which provides the thermodynamic driving force for oligomerization [5,6]. The hallmark of coiled-coils is the distinctive packing of amino acid side chain from one helix (knob) into a space surrounded by four side chains (hole) of the other helix named as ‘knobs-into-holes’. The packing in a coiled-coil interface is exceptionally tight, with almost complete Van der Waals contact between the side chains of residues at ‘a’ and ‘d’ positions. The charged residues at ‘e’ and ‘g’ positions also have an important role in the stability of the coiled-coils. Altogether, the set of interactions observed between ‘a’, ‘d’, ‘e’ and ‘g’ positions of the heptad repeat forms the basis for knobs-into-holes packing in coiled-coils [7].

The coiled-coil structures can be broadly grouped into short-length and long- length coiled-coils. The short-length coiled-coils mostly act as dimerization domains in transcription factors. The long-length coiled-coils, which consist of several hundreds of amino acid residues, are found in variety of proteins like intermediate filaments, myosins, kinesins and SMC proteins [8]. The non-ideality of the heptad register is a common fact seen in most of the long-length coiled-coil proteins like myosins and this could include either the presence of heptad breaks or the presence of unfavorable amino acids at the specific position of the heptad [9,10]. These irregularities would lead to the deviation of the perfect knobs-into-holes packing of the coiled-coil domain. In addition to the packing at the core positions of a coiled-coil, the distribution of selected hydrophobic residues at the ’a’ and ‘d’ position of the heptad is also equally important for the stability of two-stranded coiled-coils [11,12]. Besides the hydrophobic packing, the contributions of long-range electrostatic interactions also play a central role in the overall stability of coiled-coil domains [13,14]. The strength of interaction between the coiled-coil dimer is crucial, since most of them act as oligomerization units in protein structures. Regions with different strength of interactions need to be identified since they could be biologically relevant. Specificity in coiled-coil heptads and their interactions play an important role in the oligomerization process [15,16]. Studies on homo- and heterotypic coiled-coils have shown the importance of individual amino acids at particular heptad positions and their contribution to the stability [17,18]. Specifically positions ‘a’ and ‘d’ are studied extensively by substituting 20 different amino acids in a de novo-designed coiled-coil model to identify the stability and the oligomerization state [19,20]. Though coiled-coils have a simple geometry forming dimers and trimers predominantly in nature, still they are capable of forming higher order structures. These complex structures are very interesting due to their structural organization, Complexes with an oligomerization state above pentamer are gaining attraction due to their barrel-like appearance and diverse function [21]. These studies show the importance of coiled-coils and the specificity at the interface which is related to the structural organization of these simple domains.

There are different methods currently available that could predict the coiled-coil forming region from sequences, based on amino acid propensities, profile-profile comparisons, pairwise residue correlations and HMM-based approaches [22-30]. Structural analysis programs like SOCKET can determine the extent of knobs-into-holes interaction at the helix interface; thus, regions of tight packing between helices engaged in coiled-coil can be identified efficiently [31]. In addition to the sequence database of coiled-coils from the genome of *Arabidopsis thaliana*[32], a relational database CC + of coiled-coil structures processed by the SOCKET program is also available to the public domain [33,34]. Detailed analysis of long-length coiled-coils, for example tropomyosin structure, would show that many regions are quite deviated from ideality which is still unexplored. In such and other examples, weak/relatively-flexible regions within the coiled-coil are associated with a biological function of the molecule. Such emerging facts provide us an impetus to develop a method to validate a coiled-coil region given the structure based on the strength of interaction between the helices forming coiled-coils. One popular manner of associating strength or stability is to attribute psuedoenergies to the system of interest [35]. The potential energy due to intermolecular interactions at coiled-coils could be viewed as a sum of individual components such as van der Waals and electrostatic intermolecular interactions, as described in our in-house program COILCHECK [36]. The gap between sequence, structure and stability of a coiled-coil molecule is efficiently bridged by the COILCHECK program, which acts as a promising tool to identify and compare weak and strong regions within coiled-coil structures.

The magnitude and nature of the interaction between the coiled-coil dimer is provided as energy per residue in COILCHECK (for details on COILCHECK method and energy range please see [36]). Since then, the program has been updated to include new features and we refer to the new version of webserver as ‘COILCHECK+’. New features include a tool for hydrogen atom fixing and hydrogen bond energy calculation to accurately describe the prevailing stabilizing interaction at the coiled-coil interface (please see details below). An additional option is provided to choose distant-dependent dielectric constant in electrostatic energy calculation. We have also analyzed newer structural entries using the updated method and standardized the energy ranges for acceptable and stable coiled-coil regions. We also report the availability of a new structural analysis algorithm, called CHAHO within COILCHECK + webserver, for the presence of charged residue patches and ‘hydrophobic ladder’ within given coiled-coils. Finally, we illustrate the application of COILCHECK + on examples of long length coiled-coils for which biophysical studies are emerging to show their relative instability.

## Findings

### Benchmarking study of COILCHECK+

**Figure 1:**
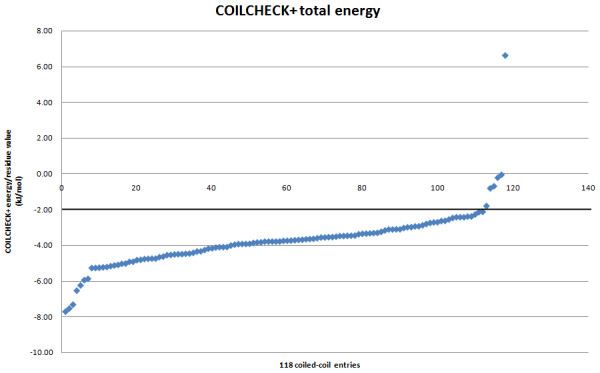
**Energy per residue for the coiled-coil dimers taken up for the current analysis.** The structures used for the study are regular coiled-coils with identified knobs-into-holes packing. A set of 118 structural entries were used for the analysis (energies are sorted from smallest to largest). The energies of 95% of these structures fall below -kJ/mol. Any coiled-coil structure with an energy better than - kJ/mol would be a stable coiled-coil, thus COILCHECK + energy could be used as a validating tool to identify the stability of the dimer

A dataset of 126 coiled-coil dimers were collected from PDB [37] and CC + database [34] as on September 2010. The collected structures were filtered using cd-hit program [38] to remove 99% identical sequences from the dataset, this resulted into a number of 112 PDB entries. All the structures were parsed through SOCKET program [31] so that regions satisfying the knobs-into-holes interactions alone could be considered for further analysis. For some of the long coiled-coil structures, SOCKET had predicted separate regions which form coiled-coils. In those cases individual PDB entries were divided into separate parts (see Additional file 1). Finally, a total of 118 structures were used for all the analysis. COILCHECK + was tested on these 118 structural entries. Hydrogen-bond, electrostatic and Van der Waals energies were calculated and were normalized as energy per residue values. The total energy per residue values are plotted for the 118 structures (Figure 1). The total energy for 95% of the structures are below -kJ/mol, thus any structure below this energy value could be a stable coiled-coil. Since all the structures analyzed are regular coiled-coil domains, this energy could be used as standard value to validate coiled-coils. We have used SOCKET processed coiled-coil domains to standardize the energy values, this is done to avoid the inclusion of regions which do not have proper knobs-into-holes interaction. Yet, we have tried to compare COILCHECK + energies of the 112 structural entries before and after parsing through SOCKET (Table 1). In general, the energies are better (energies become more negative) after parsing through SOCKET. COILCHECK + energy ranges are standardized with coiled-coils which have proper knobs-into-holes interactions, thus this adds value to the method in identifying stable and variable regions in coiled-coils. The split-up energy values for the three major energy components were compared between COILCHECK and COILCHECK+. The energies show difference in COILCHECK and COILCHECK + which is evident due to the implementation of newer set of options like hydrogen-fixing, hydrogen bond energy and DDD electrostatic energy. (please see Additional files 2, 3, 4).

**Table 1:** COILCHECK + energies before and after SOCKET for the 112 structural entries

**PDB id**	**COILCHECK + energy before SOCKET (kJ/mol)**	**COILCHECK + energy after SOCKET (kJ/mol)**	**PDB id**	**COILCHECK + energy before SOCKET (kJ/mol)**	**COILCHECK + energy after SOCKET (kJ/mol)**	**PDB id**	**COILCHECK + energy before SOCKET (kJ/mol)**	**COILCHECK + energy after SOCKET (kJ/mol)**
1A92	0.01	-0.23	1KDD	5.95	6.62	2C9N	-0.16	-4.55
1A93	9.22	-4.18	1KQL	-2.87	-3.57	2CCE	-2.43	-3.13
1 AM9	-4.54	-6.55	1L8D - 1	-1.18	-4.28	2CH7 - 1	-2.54	-2.44
1C1G - 1	-3.41	-3.34	1L8D - 2	nil	-3.93	2CH7 - 2	nil	-2.44
1C1G - 2	nil	-3.85	1LR1	-6.30	-3.04	2D3E	-4.18	-4.64
1C94	-2.29	-2.14	1N6M	-0.60	-3.88	2DFS	-0.60	-2.40
1CI6	-3.33	-4.36	1NKN	-3.83	-3.71	2DQ0	-1.23	-4.83
1CII - 1	-2.83	-5.24	1NO4	-4.70	-5.03	2DQ3	-1.45	-5.27
1CII - 2	nil	-7.73	1NWQ	-3.88	-3.67	2E4 2	-2.44	-3.80
1CII - 3	nil	-3.18	1OV9	-5.15	-1.81	2E4 3	-2.62	-3.65
1D7M	-4.99	-4.76	1PL5	-4.23	-3.80	2E7S	-2.81	-4.94
1DEB	-3.40	-4.47	1PYI	-2.27	-3.36	2EFR	-4.51	-4.51
1DH3	-2.72	-3.66	1QP9	-2.05	-3.48	2FXM	-3.65	-4.20
1DIP	-1.08	-2.64	1R05	2.22	-3.57	2GD7 - 1	-4.65	-5.11
1ECM	-5.46	-4.56	1R48	-8.10	-3.76	2GD7 - 2	nil	-5.23
1FMH	4.76	-7.33	1R6F	-5.31	-4.11	2HAP	-2.08	-7.55
1FOS	7.89	-5.28	1T2K	0.02	-2.73	2HV8	0.02	-3.73
1FU1	-1.17	-3.39	1T6F	-4.52	-4.78	2IC9	-2.12	-4.84
1GD2	-2.65	-2.99	1TMZ	-2.76	-2.40	2JEE	-3.30	-3.75
1GK4	-3.57	-3.13	1TU3	0.05	-3.49	2 K48	-3.57	-6.25
1GK6	-3.74	-4.12	1U0I	-3.73	-4.52	2OQQ	-5.52	-5.96
1GMJ	-2.22	-3.76	1UII	-4.58	-4.77	2OTO	-1.61	-2.44
1 H88	-1.67	-3.62	1UIX	-0.23	-3.84	2PMS	-0.09	-3.80
1H8A	-2.16	-3.12	1UJW	-0.58	-2.95	2Q6Q	-3.73	-3.97
1HF9	-4.06	-4.93	1UO5	-2.69	-3.26	2Q8V	-5.99	-3.80
1HJB	-1.92	-2.96	1VP7	-1.61	-2.28	2R2V	-2.00	-3.00
1I49	-1.44	-2.65	1W5I	-3.85	-2.89	2V0O	0.13	-4.12
1IC2	-4.33	-3.53	1WU9	-4.15	-5.05	2 V71	-2.31	-3.94
1IHQ	-0.01	-4.02	1X79	-0.58	-3.47	2W6A	-3.63	-3.70
1IK9	-1.97	-3.12	1YSA	-0.61	-3.94	2YSU	-0.64	-4.35
1IO4	-1.55	-2.57	1ZIL	-4.13	-2.76	2Z0O	-0.50	-3.47
1J1D	-0.65	-4.43	1ZME	-3.16	-3.37	2Z5H	-3.13	-3.55
1JCC	-3.19	-2.72	1ZWW	-1.99	-3.55	2ZTA	-3.87	-3.93
1JNM	-2.85	-4.48	2AHP	9.81	-3.81	3BBP	-1.73	-2.82
1JOC	-2.73	-3.32	2AZE	-5.00	-5.89	3BJ4	-2.76	-5.14
1JU N	7.35	-2.48	2B5U	-1.32	-4.76	3BRV	-1.05	-2.15
1K1F	-4.32	-3.33	2B9C	-1.64	-0.06	3C98	-2.98	-3.49
1KD8	5.45	-5.28	2BNI	-0.23	-4.14	3E1R	-3.65	-4.67
1KD9	4.61	-5.18	2C9L	-0.16	-4.51	3MTU	0.00	-0.82
						3MUD	-0.55	-0.70

To illustrate that the COILCHECK + energies are synchronous with energy calculations obtained using molecular mechanics and also to show that COILCHECK energies do reflect the overall quality of the coiled coil structures, we have compared the energies of few structures at different iterations of minimization process against its corresponding COILCHECK + total stabilizing energy. These structures were minimized using Tripos force field (SYBYL 7.1, Tripos Inc) and energies were calculated at ten different minimization steps (10–100 iterations). Minimization was carried out using Powell’s gradient with non-bonded interaction cut-off value of 8 and a distance-dependent dielectric constant equal to 1. Initial optimization was done using Simplex method and minimization was terminated at a convergence of 0.05 kcal mol Å^-1^. The comparison between SYBYL and COILCHECK + energies are shown in Additional file 5. There exists a high correlation between both the energy values. This shows that COILCHECK + energies are sensitive enough to reflect the minute structural changes incorporated during the minimization process.

### *CHAHO* algorithm for structural analysis of charged residue patches and hydrophobic ladder

**Figure 2:**
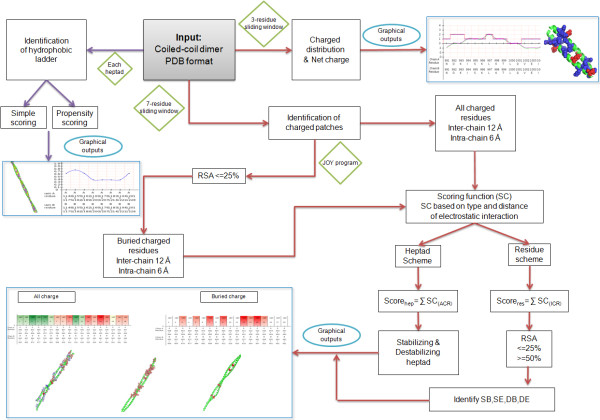
Complete protocol of hydrophobic ladder and charged-patch algorithms

CHAHO algorithm incorporates a two-pronged approach to identify clusters of stabilizing and destabilizing spatial charged-patches and to follow the continuity of the core hydrophobic ladder at the interface regions of a coiled-coil dimer. The detailed protocol of the algorithm is shown in Figure 2.

### Clusters of charged residues and their role in stability

The charged residues and long-range electrostatic interactions between these residues additionally contribute to the stability of coiled-coil dimers. Thus, using the charged-patch scoring scheme, we were able to identify stabilizing and destabilizing heptads which may have a significant role in the stability of the dimers. For the known crystal structures, where the coiled-coil boundaries are identified using SOCKET, the average charged-patch score was calculated. Most of the structures had stabilizing charged-patch score, while a few had destabilizing score (see Additional file 6). Since the charged-patch scoring scheme reflects the variable distance shell and the specific type of interaction between the charged residues, we anticipate that there will be a proper correlation between the charged-patch scores and the electrostatic energy of the proteins. Structures with a net positive score for charged-patch means that they contain larger number of favorable electrostatic interactions, thus we expect them to have good COILCHECK + energy and an inverse correlation between charged patch score and COILCHECK + energy. In order to inspect the level of relationship between these two factors, the charged-patch average score was calculated for all the analyzed structures according to the process explained in the methodology and the electrostatic energy by applying Coulomb’s equation. Ideally, higher the charge-patch score the lower the COILCHECK + energy would be. Thus, we expect negative correlation between these two values. The charge-patch score and COILCHECK + total energy/residue values for the structures analyzed are shown in Additional file 7. The correlation between these two values is negative, thereby suggesting that the method is sensitive in indentifying the stabilizing and destabilizing regions. It also emphasizes the high contribution of charged residue (electrostatic) interactions in contributing to the total energy of the system.

### Packing, continuity and specificity of core hydrophobic residues

**Figure 3:**
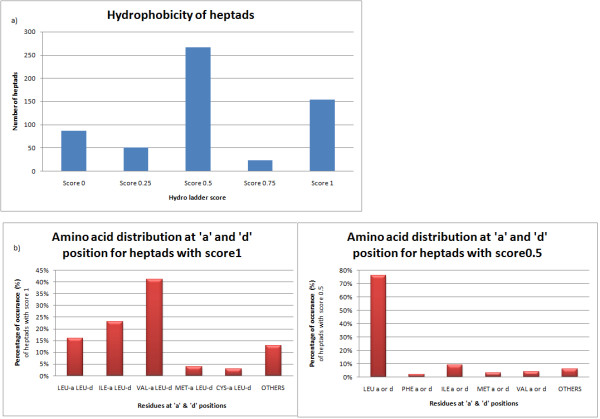
**Hydophobic-ladder analysis on the 118 coiled-coil entries.** All the structures were divided into different heptads based on SOCKET prediction. Each heptad was scored based on the residues present at a, d, a’ and d’ positions. **a**) Number of heptads assigned to respective group of hydrophobic ladder score based on the simple scoring scheme of the algorithm. **b**) Distribution of different combinations of hydrophobic residues at score 1 and score 0.5 using hydrophobic ladder scoring scheme

We had conducted an explicit study on individual heptads from 118 structural entries of coiled-coil dimers, for which the heptad positions were identified by the SOCKET program. A total of 13116 residues were examined for their specific positions and in particular the 'a' and 'd' positions were closely investigated for their amino acid preferences. An examination of the distribution of amino acids at 'a' and 'd' heptad positions (please see Additional file 8) reveal a clear preference for Isoleucine and Valine at 'a' position and Leucine at 'd' position, which is consistent with the previous study [11]. Additionally, Leucine is also found to be preferred at 'a' position and Alanine to some extent at 'd' position. Each structure was scored by the ‘hydrophobic-ladder’ program based on the amino acid propensity at the a, d, a', d' positions at the interface region (see Additional file 8 and Methods for description on scoring of hydrophobic ladder). It can be seen that the heptad scores are variable, which shows that coiled-coil dimers do not contain only hydrophobic residues at the interface core positions but other polar residues are also present. Thus, 580 heptads from 118 coiled-coil dimers were analyzed in detail for their preference of particular amino acid residues and hydrophobic ladder score. From Figure 3a, it is obviously seen that heptads with two out of four positions occupied by hydrophobic residues are found to be more dominant, followed by heptads with all four positions occupied by hydrophobic residues. This has urged us to study the amino acid combinations at the core regions (a, d, a' and d' positions) which are responsible for the zipping up of a coiled-coil dimer. Figure 3b summarizes the amino acid connections for particular heptad score. Heptads with hydrophobic residues at all four positions are dominated by Val-a;Leu-d combination. Such excellent packing of the core hydrophobic residues can be seen in DNA-bound bZIP transcription factors and human vimentin structures [39,40]. Heptads with hydrophobic residues at two of four positions (a, d, a’ and d’) are dominated by Leu at ‘a’ or ‘d’ combination. Asn-a;Leu-d pairing is found to be the most prominent of about 50% in Leu 'd' category (Additional file 9). A considerable number of heptads have polar residues, making it evident that the coiled-coil proteins are also present in solvent-exposed environments, where preferred amino acid exchanges are seen, but these heptads are less stable than the ideal ones. The score seems to be variable with each structure and for structures where the ladder score is very less. It is also coupled with very low stabilizing energy between the interfaces.

### Regions of weak interactions identified by COILCHECK+

Apart from the various programs for predicting the coiled-coil forming region from sequence data, SOCKET is the only program that uses structural information to identify coiled-coils. Despite the apparent sequence level of simplicity in retaining a regular heptad pattern, coiled-coils display considerable degree of structural diversity which leads to the formation of parallel, anti-parallel and higher order coils. These structurally distinct features have been recognized using the straightforward approach of identifying the characteristic knobs-into-holes side chain packing at the coiled-coil interface [31]. COILCHECK package further examines the strength of interactions at coiled-coils and pinpoints the weak and strong regions [36]. Although COILCHECK + is not a coiled-coil locator program, it has wide area of applications in validating the strength of interacting dimers. Regions with relatively low stability of the coiled-coil interface could be vitally required for the function of the protein. For instance, these regions could be protein-protein interaction zones or it could be a nucleation point for conformational changes that are crucial for the biological function. Here, we show the advantage of using COILCHECK + in some examples of the long length coiled-coils; though these proteins have a coiled-coil structure with regular packing, still they have stable and variable regions for a functional role. These stable and unstable regions are not identified by other coiled-coil analysis programs. The identification of ‘unstable’ dimer region in coiled-coils can pave way to explore structural importance of this simple domain which is involved in a wide variety of cellular processes.

**Figure 4:**
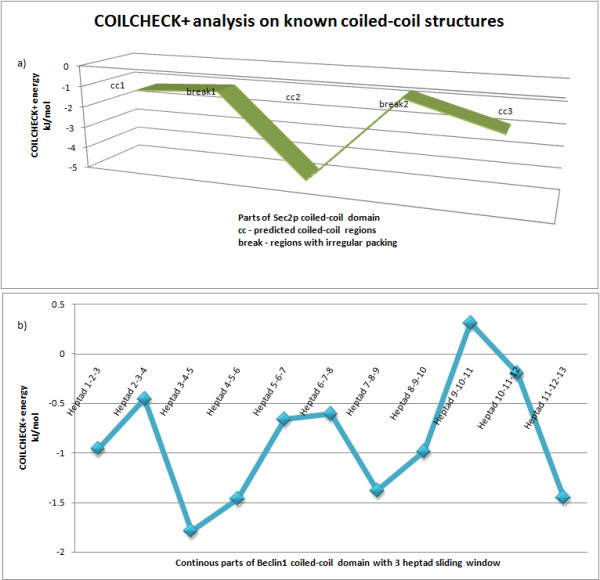
**COILCHECK + analysis on known long-length coiled-coil structures and structures belonging to DNA binding zipper class proteins. a**) COILCHECK + energies of different parts of Sec2p coiled-coil domain is shown. The structure (PDB id 2OCY) is divided into 5 parts based on SOCKET coiled-coil prediction and COILCHECK + energy for each part is identified. It can be clearly seen that the coiled-coil regions are assigned good energies and the break regions have poor energies. **b**) Beclin1 coiled-coil domain (PDB id 3Q8T), which has continuous heptad pattern, is divided into overlapped three heptad parts and each structure is checked for its COILCHECK + energies. It can be visualized that all the regions are less stable and it also couples with the irregular a-d pairing reported by Li and coworkers.

The first example is a catalytic Sec2p GEF domain (PDB id:2OCY) which contains coiled-coil. The structure is a parallel homodimeric 220Å long coiled-coil domain which is kinked near the N-terminus due to the presence of a stammer. The hydrophobic packing at the core region is also disturbed between residues 94 and 119 and it is shown that this region is a binding site for Sec4p [41]. SOCKET program identifies three regions that form proper coiled-coil with ideal knobs-into-holes interactions. Thus, the structure was divided into five parts: three coiled-coil regions which were identified using SOCKET and two breaks. COILCHECK + was used to identify the energies for these five parts of the structure (Figure 4a). The regions of higher stability in the coiled-coil regions can be recognized with good energy when compared with the breaks.

The second example is an anti-parallel coiled-coil domain of beclin1 protein (PDB id: 3Q8T). The protein is composed of a series of imperfect a-d pairing at the interface. The coiled-coil dimer domain is shown to be metastable at physiological temperatures using circular dichroism studies and the metastable property of this homodimeric coiled-coil domain is required for its ready transition to the more stable and functional beclin1-Atg14L/UVRAG heterodimer [42]. SOCKET identifies this structure as a proper coiled-coil domain with complete heptad pattern. But the stability of the coiled-coil domain which is related to the function of the protein need to be identified, this is efficiently done by COILCHECK+. The overall COILCHECK + energy for the structure 3Q8T is -1.161 kJ/mol which is quite low showing that the coiled-coil domain is less stable. To gain further details on each heptad and variable regions, the 13-heptad full length structure was divided into eleven parts, where each part has three heptads and one heptad has a overlap with the previous structure. Each of the structure was analyzed using COILCHECK+. The energy values are shown in Figure 4b, it can be clearly seen that all the parts have energy above - kJ/mol and certain regions were very variable. These less stable heptads (heptad-3,5 &6) are regions where the imperfect a-d pairing resides and the unfavorable repulsive pair E224a-D221e’ is present in heptad7 which is also found to be less stable. Li and coworkers have shown that these imperfect residue interactions are responsible for the metastable beclin1 homodimer. Additionally, instability is caused by clustered negatively charged residues near heptad- 3 and 10, long range electrostatic interactions between these ionic residues (E184, E188, E189, E190, E240, D242, D243, E244) provide large amount of unfavorable repulsive energy to the system. These unfavorable electrostatic interactions could be another factor that contributes to the lower stability of the coiled-coil domain.

The third example the tropomyosin mid-region (PDB id: 2B9C). Since tropomyosin is wound around the F-actin polymer, it should have flexible zones which would permit the coiled-coil to bend and bind with the actin. The middle region of tropomyosin coiled-coil domain is detected to be flexible which is responsible for the bending of the molecule [43]. Though the structure is identified to be a proper coiled-coil with complete heptad pattern, COILCHECK + energy for this flexible mid-region is very poor (0.13 kJ/mol), showing the unstable nature of this region.

COILCHECK + was able to distinguish the relatively unstable flexible regions that could provide biological importance and value. Such examples also show the importance of bridging the gap between identification and validation of coiled-coils. It is shown that identifying the relatively unstable regions would also throw light in the direction of recognizing functionally important regions which is biologically relevant.

### Analysis on specific systems

#### Study on a well-known structure: tropomyosin

**Figure 5:**
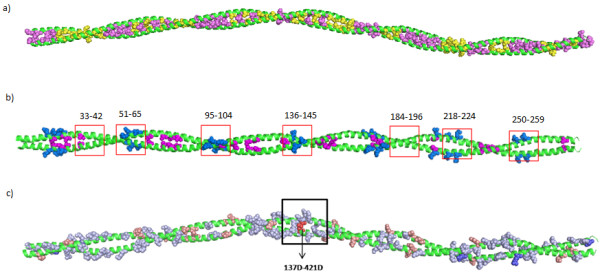
**The results of CHAHO algorithm for tropomyosin structure (1C1G). a**) Output of hydrophobic ladder shows the presence of alanine residues at the core positions are responsible for the flexibility of the protein (alanine staggers-yellow spheres, hydrophobic residues-magenta spheres). The hydrophobic ladder has eight specific breaks. **b**) The method was able to identify negatively charged residue patches on the structure which could help in interacting with actin (Magenta spheres-alanine clusters, Blue spheres-actin binding sites, Red box-predicted hot spot and the residue ranges for each of the boxed region are shown). **c**) 1C1G structure mapped with SB, SE, DB and DE residues (SB: stabilized buried charged patches, please see text for abbreviations). Boxed region shows the destabilizing negatively charged pairs which could be responsible for the bending of mid-region in the tropomyosin structure.

Tropomyosin is a long coiled-coil protein which overlaps and binds end-to-end with the adjacent tropomyosin molecule [44,45] and wraps around the entire length of actin filament. This cooperative binding of tropomyosin with actin is mediated by unique sequence features; this can be seen as sharp bends, which is due to alanine clusters at the core positions and the seven-fold periodicity of charged residues which span the entire structure [46,47]. Since tropomyosin has been one of the well-examined coiled-coil structures since 1960's, a detailed and careful analysis on this system would add value to the developed methods. Here, the full-length crystal structure of tropomyosin 1C1G with 40 heptads was used for the analysis of the developed programs. The breaks in hydrophobic ladder could be clearly observed due to the presence of alternating hydrophobic and other residues. This sequence-specific feature marks an important factor for the flexibility of tropomyosin structure. Indeed, the hydrophobic ladder was disrupted eight times due to the presence of non-hydrophobic residues which also includes the alanine staggers (Figure 5a). It is also known that a single tropomyosin molecule binds to seven F-actin monomers. Since both actin and tropomyosin are abundant in solvent-exposed charged residues, it is reasonable to assume that the tight binding between them is mediated by electrostatic interactions. The seven-fold periodicity of negatively charged residues on the surface of the tropomyosin molecule is indeed responsible for its interaction with the actin filament [48,49]. CHAHO suite was able to successfully identify solvent-exposed spatial-charged clusters on the structure which might be involved in interactions with the tropomyosin and actin binding regions due the higher solvent accessible residues. The method was also able to identify stabilizing and destabilizing heptads on the structure and map the charged residues which are important for the stability of the coiled-coil dimer (Figure 5b).

Charged residues were classified into four major categories on the basis of their solvent accessibility and charged-patch score. The list of these important residues is given in Table 2. When only buried charged residues were considered 15 K-A, 137D-A, 218D-A and 421D-B were identified as buried destabilizing residues and 299 K-B and 502E-B as partially buried destabilizing residues. The effects imposed by the clustered and isolated core alanine residues play an important role in the flexibility and bending of tropomyosin coiled-coils. The local instability caused by the isolated A134 in the midst of apolar core residues would permit variable bends [43]. In addition to the isolated alanine at this region, it was identified that the instability is caused by the buried destabilizing negatively charged pair 137D-421D which is seen to be the adjacent core residue next to 134A-418A and located near the large apolar core side chains (Figure 5c). Earlier study shows that aspartic acid at the core positions disrupts parallel coiled-coil and creates a flexible hinge [50]. Hence, the high unfavourable electrostatic energy produced by this destabilizing pair found in the core region would play a crucial role in further instability and bending of the tropomyosin coiled-coil segment in the middle. Thus, the study of tropomyosin coiled-coil using the hydrophobic ladder and charged-patch programs had yielded valuable information on specific residues which are vital for the structure and function of the protein.

**Table 2:** Classification of charged residues of 1C1G tropomyosin structure into four categories: SB (stabilizing buried), SE (stabilizing exposed), DB (destabilizing buried), DE (destabilizing exposed)

**SB**		**SE**								**DB**		**DE**	
**A**	**B**	**A**				**A**				**A**	**B**	**A**	**B**
15 K	313 K	2 D	66 D	152 K	272 E	296 K	375 R	436 K	524 E	137 D	421 D	20 D	291 E
218 E		5 K	72 E	168 K	276 H	300 E	380 E	440 E	527 E			56 E	307 D
244 R		12 K	77 K	178 R		305 R	389 R	461 E	528 R			80 D	339 D
280 D		16 E	91 R	182 R		312 D	396 K	462 R	534 E			84 D	340 E
		21 R	112 K	189 K		314 K	398 E	464 E	538 D			100 D	342 D
		23 D	114 E	213 K		318 D	399 E	466 R	543 E			104 D	353 E
		24 E	117 E	217 K		321 K	401 E	471 E	558 D			150 E	364 D
		28 D	118 K	219 D		324 E	402 K	482 K	559 H			164 E	368 D
		30 K	121 D	220 K		325 D	408 E	497 K				181 E	382 E
		37 K	124 E	226 K		333 K	412 K	503 D				192 E	384 D
		40 E	125 R	230 D		335 K	417 R	504 K				196 E	406 E
		41 D	128 K	234 E		343 K	420 K	508 E				198 K	437 H
		48 K	131 E	236 E		446 E	422 E	514 D				223 E	443 D
		55 D	133 R	238 R		354 K	423 E	515 K					476 E
		58 D	142 E	248 K		359 E	424 K	518 E					478 E
		59 K	145 E	251 K		360 K	429 E	520 E					500 K
		65 K	149 E	264 K		361 K	433 K	522 R					552 K

#### Myosin VI medial tail and its importance

Myosins are diverse family of actin-based molecular motors. The general structure of myosins consists of a N-terminal head domain followed by neck domain of variable lengths and the C-terminal domain which has the coiled-coil and the cargo-binding domain. Different biological functions within this large family of molecular motors depend on the differences in their tail domains, commonly rich in the α-helical coiled-coil motif. Myosin VI is a unique class of unconventional myosins that has a reverse directionality on actin movement [51]. In spite of having a short neck region, the molecule takes up ~36 nm step size and acts as a processive molecule travelling long distances along the actin filament [52]. The myosin VI tail is composed of four distinct domains: the proximal tail (PT), medial tail (MT), distal tail (DT) and the cargo binding domain (CBD) [53]. The medial tail has gained importance in the recent past, where different views are put forward to address the structure and function of this region. The study of Spink and coworkers had showed that dimerization happens at the CBD and the MT domains, with clusters of charged residues acting as a single α-helix, stable enough to bridge the gap between the two heads in a 36 nm step. It is also noted that the MT is composed of ER/K motif which gives substantial rigidity to the α-helical region [54]. Another study has showed that the PT region adopts a three-helix bundle conformation and the dimerization is initiated by two CBD regions at close proximity. Since the initial part of MT region has properly spaced hydrophobic residues, this region may be engaged in forming a coiled-coil, leading to the unfolding of PT and the lever arm extension is responsible for the large step of the molecule [55]. The fact that MT region is strongly predicted to form coiled-coil by various programs like PAIRCOIL2 and COILS and the region has clusters of charged residues has urged us to study the human myosin VI MT with our developed methods to gain insight on its functional importance.

**Figure 6:**
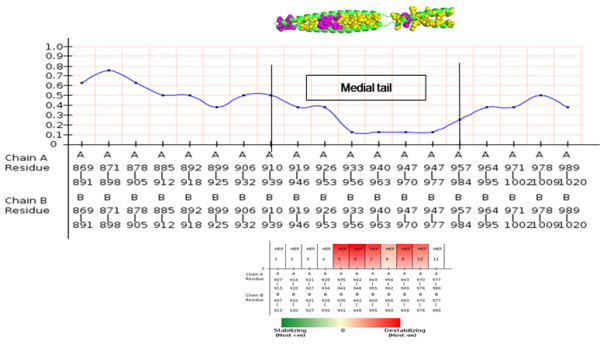
**Hydro ladder and charge patch results for myosin VI medial tail.** Hydrophobic ladder score plotted with medial tail region mapped with hydrophobic (magenta spheres) and non-hydrophobic (yellow spheres) residues and the destabilizing heptad identified using the buried charged-patch protocol for the medial tail is shown in parallel. A 5-heptad sliding window has been used for the calculations and for this graphical representation

The MT of human myosin VI sequence was modeled based on PAIRCOIL2 heptad prediction with an ideal coiled-coil structure as template (please see Additional file 10). The MT model was analyzed using hydrophobic ladder and charge-patch algorithms and COILCHECK + energies were used to understand the significance of this region. The hydrophobic ladder scores were relatively low at the MT region, when compared to the whole region predicted as coiled-coil (Figure 6). The charged-patch program, with all charge and buried charged residues could identify both stabilizing and destabilizing heptads, along with the crucial residues which may have functional importance (Table 3). The modeled structure was also analyzed using the COILCHECK + server, where we find the MT region to be weakly stable with poor energies. The stability of the truncated construct (908–940 residues), which was speculated to form coiled-coil by Mukherjee and coworkers [55] was also analyzed separately, where this region also had poor energies making it an unstable coiled-coil (Table 4).

**Table 3:** Charged residues of human myosin VI MT (medial tail) are classified into SB (stabilizing buried), SE (stabilizing exposed), DB (destabilizing buried) and DE (destabilizing exposed) categories along with buried DB (destabilizing buried) residues

**SB**		**SE**				**DB**		**DE**				**Buried DB**	
**A**	**B**	**A**		**B**		**A**	**B**	**A**		**B**		**A**	**B**
943 D	943 D	916 K	946 R	925 R	959 E	922 E	922 E	915 K	952 E	915 K	955 R	936 E	936 E
957 K	957 K	917 K	953 E	927 R	969 E	936 E	936 E	924 E	955 R	916 K	965 K	943 D	943 D
964 R	964 R	925 R	959 E	935 K	972 K	950 E	950 E	931 E	965 K	920 E	977 D	950 E	950 E
967 E	970 E	928 R	972 K	937 R	973 K		967 E	934 E	975 E	921 E	978 E	967 E	957 K
970 E	974 R	935 K	976 D	941 E	975 E			940 R	977 D	924 E	979 K	970 E	967 E
974 R		937 R		942 E	976 D			941 E	979 K	934 E			970 E
		938 K		948 R				948 R		946 R			974 R
		942 E		952 E				949 K		949 K			
		945 K		953 E				951 E		951 E			

**Table 4:** COILCHECK + energies of medial tail and 908–940 modelled construct

**Energies (kJ/mol)**	**Medial tail**	**908-940 Construct**
H-bond	-39.999	-3.405
Electrostatic	81.073	129.875
van der Waals	-352.450	-131.162
Total	-311.376	-4.692
Eng/Res	-2.100	-0.070

The entire study on human myosin VI medial tail has shown that the two helices, assumed to form coiled-coil, are very poorly stabilized with each other due to the lack of hydrophobic residues at the core position throughout the complete stretch. Although few hydrophobic residues are present in the initial part of the tail, it appears that the repulsive electrostatic interactions would not permit the helices to form a stable coiled-coil. The dimerization of CBD, which leads to the close proximity of the tail regions, may induce interactions between charged residues permitting them to dimerize. In a physiological state (with water and salt in the protein environment), the exposed charged residues would be shielded by salt and the interaction between the buried residues would be the sole responsible unit for dimerization. However, in the MT region of myosin VI, the presence of buried charged residues suggests that this region is highly destabilized. Even though the MT region of myosin VI is predicted to be a strong coiled-coil by various programs, our analysis and energies show that the interactions between the two helices are weak and unstable. This shows the importance of examining hydrophobic ladder and spatial buried charged clusters to gain a better understanding of the stability of coiled-coils. Likewise, regions in coiled-coil with abundant solvent-exposed spatial charge clusters could be zones of protein-protein interactions. This analysis also provides impetus to carry out detailed examination of the spatial distribution of sequence features on various proteins that contain coiled-coils.

## Conclusions

Coiled-coils are important structural motifs playing vital role in various fields of biology. It is important to have a better understanding of the interactions and energetic of the molecule which is the driving force for specific association. Regions of coiled-coils with relatively weak stability and blocks of flexible zones on the long-length coiled-coil proteins, usually coupled with imperfect heptads and/or solvent-exposed spatial charge clusters, are assumed to be potential target binding sites.

In order to understand the contribution of the different interactions to the stability of coiled-coil dimers, we had conducted an extensive structural analysis on known two-stranded coiled-coils using both SOCKET [31] and COILCHECK [36]. The results of the analysis had lead to an understanding that the inter-chain electrostatic interactions (contributed by clusters of charged residues) and the continuity of the core hydrophobic ladder (forming the ideal knobs-into-holes packing) are extremely important to the overall stability of the protein. An explicit analysis of hydrophobic ladder and the presence of unfavorable charged-patches can provide a better understanding of weakly stable or functionally important zones within coiled-coils. This has urged us to develop a dedicated algorithm which will validate the coiled-coil structure based on the energies, evaluate the distribution of spatial clusters of charged residues, to examine the continuity of the hydrophobic residues at the core positions and identify hot spot zones which are stable and destable. Such an algorithm will also act as a promising tool to identify structurally vital residues and to study many important protein families. This can also guide in designing point mutations which could reveal functional importance of pivotal residues, to distinguish particular regions in the coiled-coil motif rich in charged residues and to provide clues about protein-protein interaction sites.

We have updated our coiled-coil validation webserver from COILCHECK to COILCHECK+. The improved version includes a tool for fixing hydrogen atoms to a given PDB structure and calculating the hydrogen bond energy at the interface and an option to choose distant-dependent dielectric in the electrostatic energy calculations. Additionally, we have developed CHAHO a program to identify clustered charged residues and the continuity of core hydrophobic residues. This additional emphasis for the presence of charged residue clusters and detailed examination of the hydrophobic ladder was required in order to detect weakly stabilized regions which may be missed during average pseudoenergy calculations.

## Methods

### Geometric fixing of hydrogen atoms and calculation of hydrogen bond energy in COILCHECK+

The method utilized for fixing hydrogens is as delineated here. The position of hydrogen with respect to the connecting atom has been determined/fixed geometrically using standard bond lengths, angles and torsion angles for all types of relevant atoms to make up hydroxyl, carboxyl, methyl, methylene, tertiary groups and considering sp3, sp2, sp atomic states of hybridization following the published method [56]. The hydrogen bond energy is calculated based on Kabsch and Sander’s equation [57], where only inter-chain hydrogen bonds are considered to attribute energy values. The hydrogen bonds are categorized into three classes: interchain backbone-backbone (BB), backbone-side chain (BS) and side chain-side chain (SS) interactions. The details of the residues involved in hydrogen bonding at a defined cutoff of 3.2Å (the donor-acceptor distance), type of interaction and the energy contributed are also provided in the output.

### Distant-dependent dielectric in electrostatic energy calculation in COILCHECK+

Electrostatic energy is calculated based on Coulomb's equation. All inter-chain electrostatic interactions between the charged residues (ARG, LYS, HIS, ASP and GLU) within a cutoff of 15Å are considered for calculation of energy.

(None) $$E_{\mathrm{ele}}=\frac{4.84^{*}332^{*}q1^{*}q2}{\left(D^{*}r\right)kJmol}-1$$

The charges q1 and q2 for each charged residue were taken from CHARRM package [58], we had used distance dependent dielectric (DDD) constant in the energy calculation where D = 2r [59] and r is the distance between the two charged atoms. The use of DDD in electrostatic energy calculation is able to yield an appropriate energy value depending on the strength of interaction seen between the residues.

### Identification of spatial charged-patches

To identify clusters of charged residues which are responsible for stabilizing and destabilizing the intensity of interaction between the coiled-coil dimer, we developed a routine called ‘charged-patch’ within CHAHO. The aim of the charged-patch method is to identify specific regions or charged patches in the coiled-coil dimer which are important for the stability of the protein. Given a PDB structure and the two interacting chain identifiers, the program employs a sliding window of each heptad on both the chains and the residues are first classified into buried and exposed based on their relative solvent accessibility (RSA; identified by the PSA program from JOY package [60]) (please see Figure 2 for a complete protocol). For every individual charged residue of an heptad, the number of other interacting charged residues are identified for two distance ranges - a 6Å intra-chain and 12Å inter-chain distant shells. It is well-known that electrostatic interactions (between charged residues) can traverse fairly long distances, but become weak at longer lengths. In order to account for this, the scores are weighed according to the distance between two charged residues, variable distance shells (of 3Å, 6Å, 9Å and 12Å) were considered to add weight to the score to each type of interactions at different distances. Every charged residue is assigned a charged-patch score which ranges from zero to one based upon the electrostatic interactions with its partner at different distant shells. The program adopts a two-way approach of consolidation in which scoring function (SC) takes up the heptad and residue scheme. In the heptad scheme, the score is a cumulative value of the interactions of all charged residue (ACR) in the heptad and in the residue scheme every individual charged residue (ICR) is assigned a score based on its interacting partners and the distance shell. Thus stabilizing/destabilizing heptads and stabilizing/destabilizing residues are identified based on their scores and the set of residues forming the charged-patch for the stability of the system are also recognized.

The presence of charged patches on the surface of coiled coils could be required for interactions with other proteins. However, clusters of like-charged residues within charge patches could contribute to poor stability to a coiled-coil system. Therefore, the solvent burial of charged residues were considered and charged patches were categorized as solvent-exposed (E) or solvent-buried (B). Based on their RSA values and scores, charged residues are categorized into four groups: Stabilizing buried (SB), Stabilizing exposed (SE), Destabilizing buried (DB) and Destabilizing exposed (DE) pairs of residues. Apart from the charged-patch, which states the magnitude of interaction within the coiled-coil dimer interface, the distribution of charged residues in the sequence is also found to be structurally important, because it additionally conveys the information about the probable sites for protein-protein interactions. Hence, at a defined sliding window size, the distribution of positive and negative charged residues and the net charges are identified and displayed. Finally, the identified charged-patch at the residue and heptad levels and the distribution of charged residues are mapped on the structure. The identification of such spatial charged-patches will permit us to recognize hot spots which are crucial for the stability of the structure. This could also enable us to identify key residues that can either stabilize or destabilize the system.

### Measurement of hydrophobic content in hydrophobic ladder

The ‘hydrophobic-ladder’ score, part of CHAHO algorithm, is designed to examine the hydrophobic content of ‘a’ and ‘d’ positions of the assigned heptads at both helices that engage to form the coiled-coil. The more hydrophobic these positions are at a heptad, the higher the score will be. To begin with, we assigned a score of 0.25 if any of these four positions in a coiled-coil heptad was occupied by a hydrophobic residue (Ala/Val/Leu/Ile/Phe/Tyr/Trp) and 0 if it is not. A maximum score of 1 can be expected for a heptad. Whereas ideally ‘a’ and ’d’ positions at both helices of a heptad is expected to retain hydrophobic residues for conducive and stabilizing interactions, this may not be the case in real-time sequences. The presence of every hydrophobic residue at any of the four positions in a heptad contributes 0.25 each to the score at a heptad.

Next, we also devised a propensity-based scoring scheme to normalize for the frequency of occurrence of certain hydrophobic residues. The propensity values of the hydrophobic residues from MTIDK matrix [23] are directly incorporated to show the amino acid preferences at the particular positions. Scores were assigned to every heptad and graphs are plotted where the values are smoothened over a five-heptad sliding window. Such smoothened graphs will allow the user to identify regions which are stable and likely to be unstable, thereby providing a clue about the flexibility of the structure at particular regions.

## Availability and requirements

Project name: COILCHECK+.

Project home page: http://caps.ncbs.res.in/coilcheckplus.

Operation system(s): Ubuntu Linux 11.10+.

Programming language: Java, C++, Perl, PHP.

Other requirements: None.

License: None.

Any restrictions to use by non-academics: COILCHECK + can be used free-of-charge by non-academics, provided appropriate citation and credit is given to the authors of this publication.

The desired format of input file would be a PDB file with two chain identifiers. However, the user can select two chains out of a multi-chain PDB file as well. The COILCHECK + server calculates energies between two chains of a PDB file; ideally the parameters have been standardized with dimeric coiled-coils. Parallel/antiparallel, homodimeric, heterodimeric coiled-coils can be validated using COILCHECK + server. Additionally, user can select from different options for type of electrostatics calculation, charged-patch and hydrophobic ladder algorithm, apart from the default options. The outputs can also be selected by the user for particular energy component and visualization of different type of interacting residues are provided. The output page contains energy terms, possible interactions, charged-patch score and hydrophobic ladder score plots. The energy terms provided are hydrogen bond, electrostatics and Van der Waals components, along with the corresponding potential interactions like hydrophobic interactions and short contacts. The hydrophobic ladder score and charge patch scores are averaged over a seven-residue window and can be used best when both the chains start from equivalent corresponding heptads.

## Availability of supporting data

The data sets supporting the results of this article are included within the article (and its Additional files 1, 2 and 3).

## Abbreviations

CHAHO: Algorithm for the analysis of charged residue clusters and hydrophobic ladders; DDD: Distance dependent dielectric constant.

## Competing interests

The authors declare that they have no competing interests.

## Authors' contributions

RS designed the overall research work, MS developed the CHAHO algorithm and performed the analysis on different system, AN developed the web version. AC, KJ, SM developed hydrogen-fixing, H-bond energy calculations, DDD electrostatic energy calculation programs and the graphical java utilites. MS drafted the manuscript and RS provided critical reading and improvisation of the manuscript. All authors read and approved the final manuscript.

## Supplementary Material

Additional file 1 Details of the data set used fpr the analysis; PDB IDs of the 112 coiled-coil structures, chain IDs of the dimer, their description, resolution, residue boundaries before & after parsing through SOCKET and the orientation of coiled-coil dimer. Click here for file

Additional file 2 COILCHECK energy split-up values for structures used for the analysis. Click here for file

Additional file 3 COILCHECK + energy split-up values for structures used for the analysis. Click here for file

Additional file 4 Energy split of structures using COILCHECK and COILCHECK + webserver. Click here for file

Additional file 5 Correlation between Tripos force field minimization energy and COILCHECK + total stabilizing energy. Click here for file

Additional file 6 Distribution of charged patch score for the analyzed coiled-coil dimmers. Click here for file

Additional file 7 Correlation between the charged-patch score and total COILCHECK + psuedoenergy for known structures. Click here for file

Additional file 8 Distribution of 20 amino acids at a and d heptad positions and Hydrophobic ladder score dispersion for coiled-coil structures analyzed. Click here for file

Additional file 9 Different amino acid pairing of leucine at ‘d’ position with score 0.5 and Different amino acid pairing of leucine at ‘a’ position with score 0.5. Click here for file

Additional file 10 Modelling of Medial Tail of Human myosin VI sequence. Click here for file
