# Supplementary figures and images for: Structural attributes for the recognition of weak and anomalous regions in coiled-coils of myosins and other motor proteins
Source: BMC Res Notes. 2012 Sep 25;5:530. doi: 10.1186/1756-0500-5-530 (PMC3542152; doi:10.1186/1756-0500-5-530)

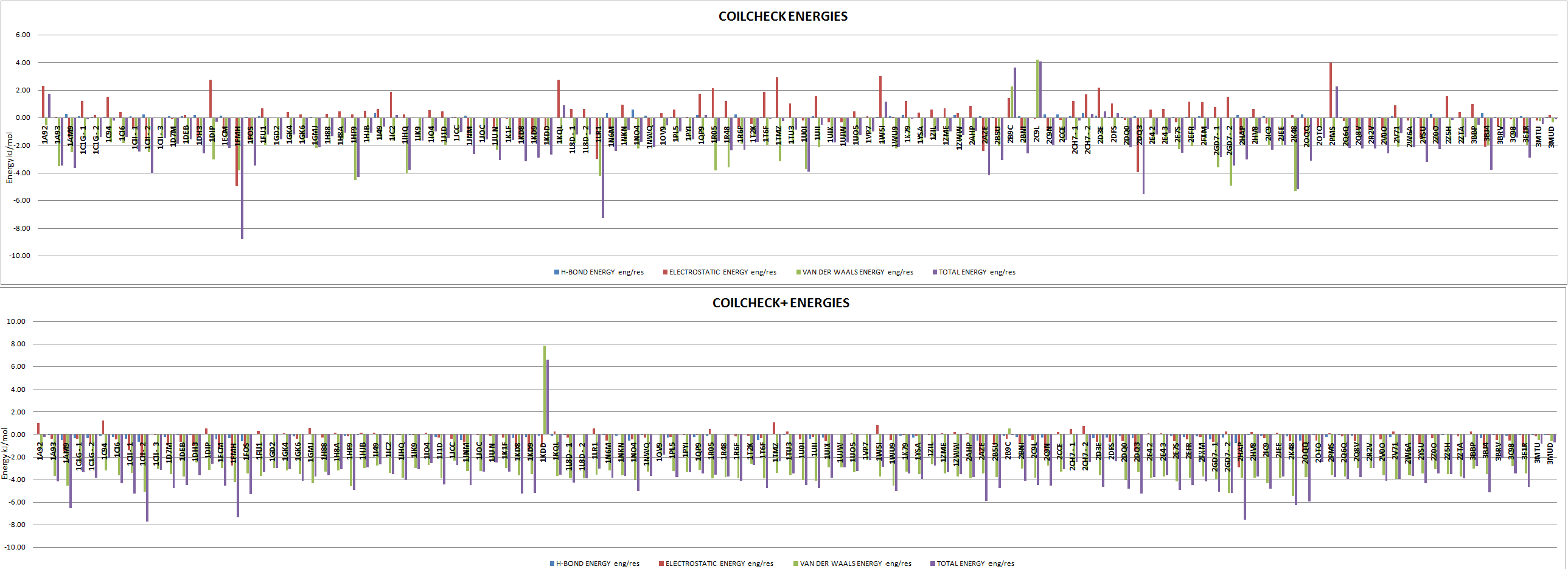

Supplement: Additional file 4 — Energy split of structures using COILCHECK and COILCHECK + webserver. [file 1756-0500-5-530-S4.tiff]

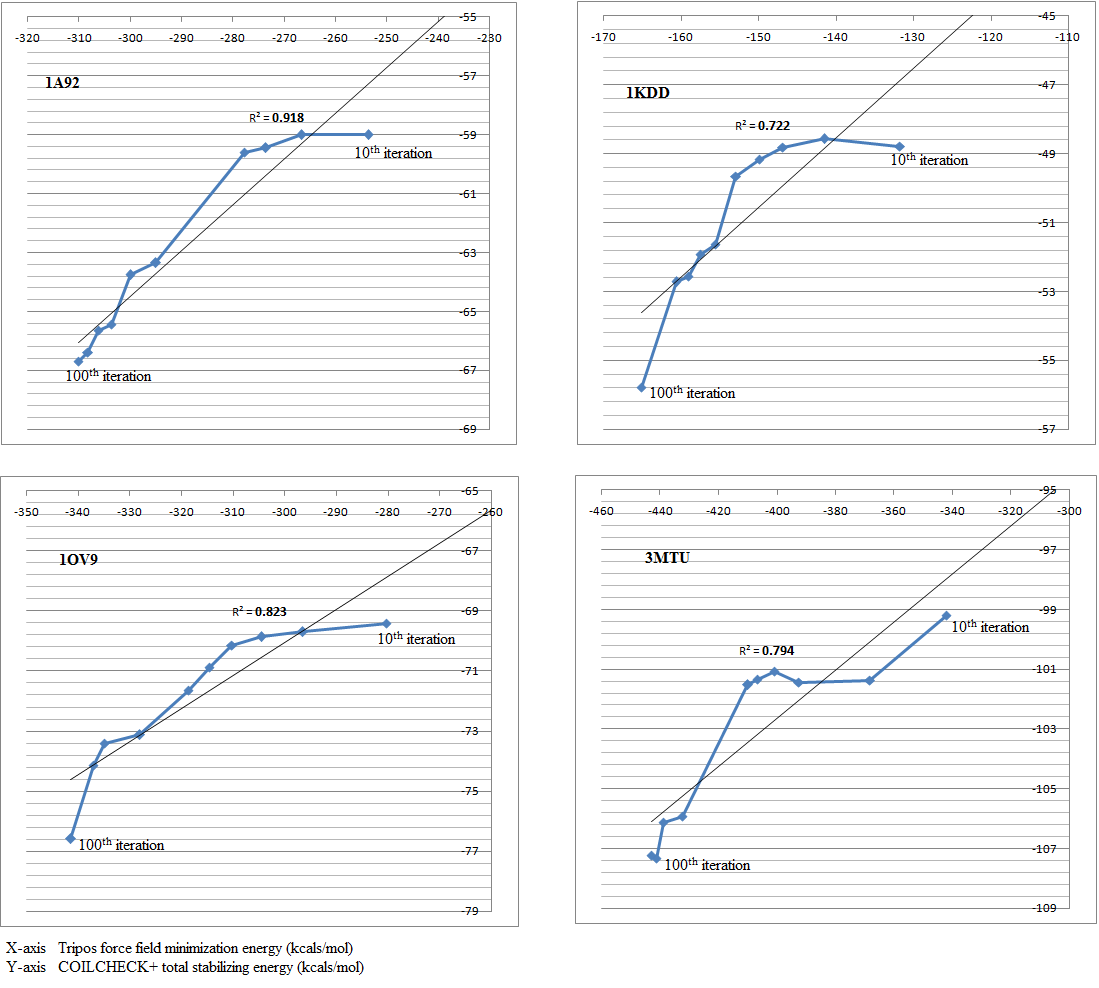

Supplement: Additional file 5 — Correlation between Tripos force field minimization energy and COILCHECK + total stabilizing energy. [file 1756-0500-5-530-S5.tiff]

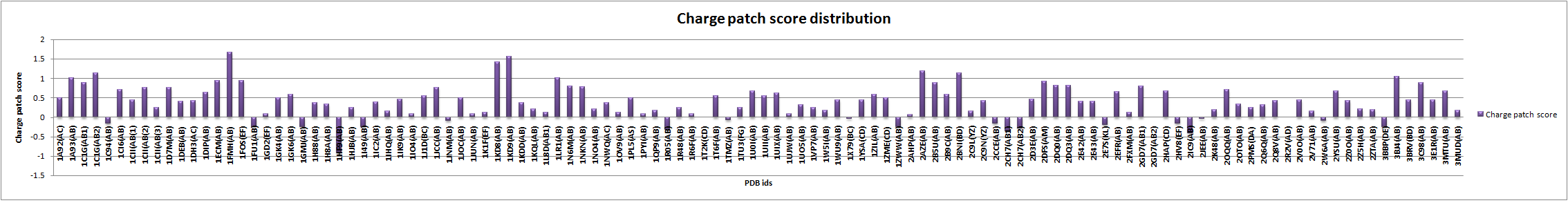

Supplement: Additional file 6 — Distribution of charged patch score for the analyzed coiled-coil dimmers. [file 1756-0500-5-530-S6.tiff]

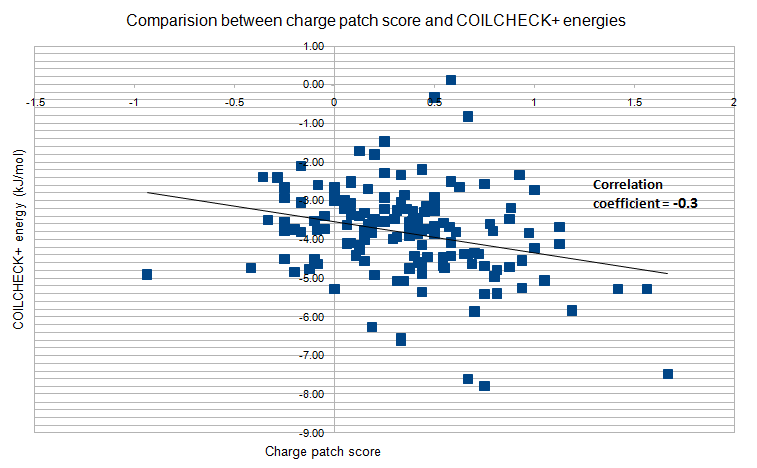

Supplement: Additional file 7 — Correlation between the charged-patch score and total COILCHECK + psuedoenergy for known structures. [file 1756-0500-5-530-S7.tiff]

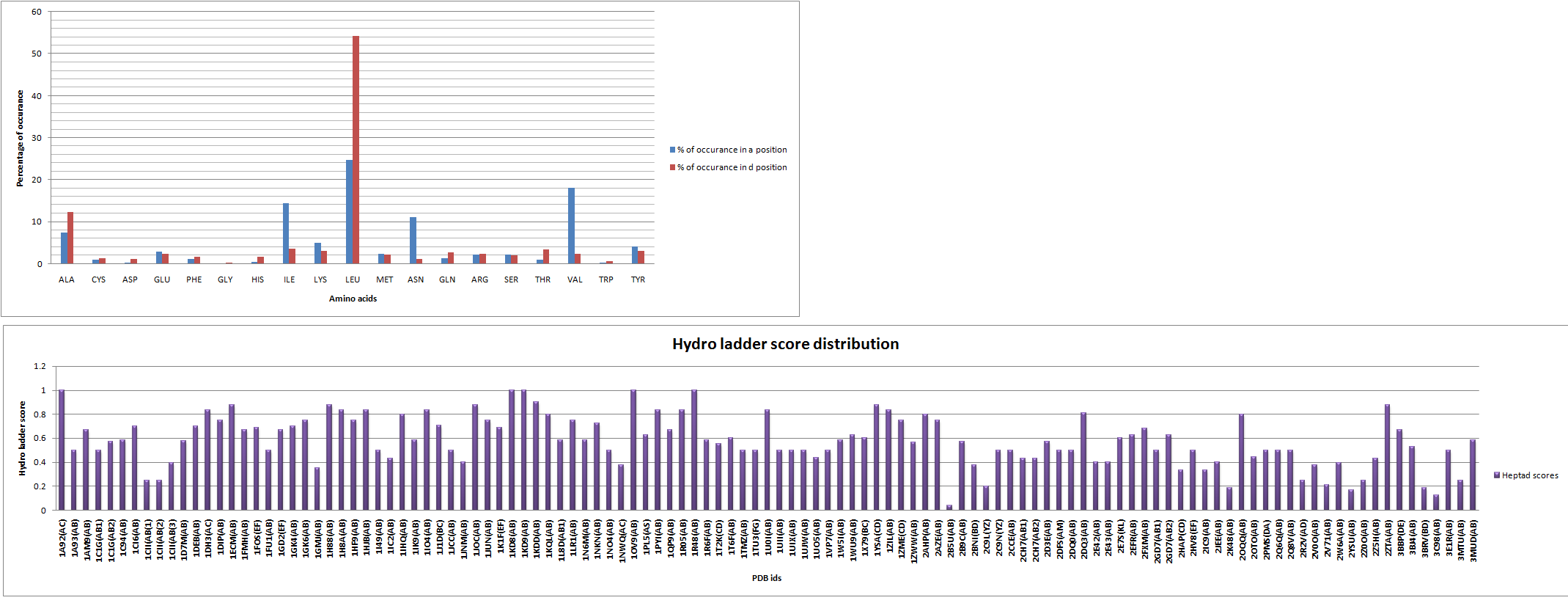

Supplement: Additional file 8 — Distribution of 20 amino acids at a and d heptad positions and Hydrophobic ladder score dispersion for coiled-coil structures analyzed. [file 1756-0500-5-530-S8.tiff]

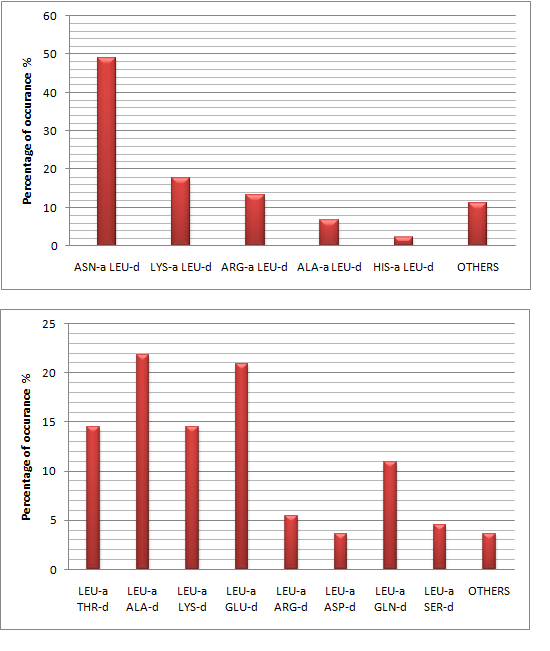

Supplement: Additional file 9 — Different amino acid pairing of leucine at ‘d’ position with score 0.5 and Different amino acid pairing of leucine at ‘a’ position with score 0.5. [file 1756-0500-5-530-S9.tiff]
