# Supplementary material for: Structural attributes for the recognition of weak and anomalous regions in coiled-coils of myosins and other motor proteins
Source: BMC Res Notes. 2012 Sep 25;5:530. doi: 10.1186/1756-0500-5-530 (PMC3542152; doi:10.1186/1756-0500-5-530)
Supplement: Additional file 10 — Modelling of Medial Tail of Human myosin VI sequence. [file 1756-0500-5-530-S10.doc]

**Modelling of Medial Tail of Human myosin VI sequence**

Template: 2EFR

Target: myosin-VI [Homo sapiens] - NP_004990.3

**Paricoil2 prediction**

2EFR (148-301)

MKQLEDKVEELLSKNYHLENEVARLKKLLERAEERAELSEGKSAELEEELKTVTNNLKSLEAQAEKYSQKEDKYEEEIKVLSDKLKEAETRAEFAERSVTKLEKSIDDLEDELYAQKLKYKAISEEMKQLEDKVEELLSKNYHLENEVARLKKL

abcdefgabcdefgabcdefgabcdefgabcdefgabcdefgabcdefgabcdefgabcdefgabcdefgabcdefgabcdefgabcdefgabcdefgabcdefgabcdefgabcdefgabcdefgabcdefgabcdefgabcdefgabcdefg

NP_004990.3 (869-1020)

KQIKNLEISIDTLMAKIKSTMMTQEQIQKEYDALVKSSEELLSALQKKKQQEEEAERLRRIQEEMEKERKRREEDEKRRRKEEEERRMKLEMEAKRKQEEEERKKREDDEKRIQAEVEAQLARQKEEESQQQAVLEQERRDRELALRIAQSE

fgabcdefgabcdefgabcdefgabcdefgabcdefgabcdefgabcefgabcdefgabcdefgabcdefgabcdefgabcdefgefgabcdefgabcdefgabcdefgabcdefgabcdabcdefgabcdefgabcdefgabcdefcdefg

The target was modelled using 2EFFR as template based on heptad position alignment.

**Target – Template alignment**

KQIKNLEISIDTLMAKIKSTMMTQEQIQKEYDALVKSSEELLSALQKKKQQEEEAERLRRIQEEMEKERKR

fgabcdefgabcdefgabcdefgabcdefgabcdefgabcdefgabcefgabcdefgabcdefgabcdefg

--MKQLEDKVEELLSKNYHLENEVARLKKLLERAEERAELSEGKSAEEEELKTVTNNLKSLEAQAEKYSQK

--abcdefgabcdefgabcdefgabcdefgabcdefgabcdefgabcefgabcdefgabcdefgabcdefg

REEDEKRRRKEEEERRMKLEMEAKRKQEEEERKKREDDEKRIQAEVEAQLARQKEEESQQQAVLEQERRDRELALRIAQSE

abcdefgabcdefgefgabcdefgabcdefgabcdefgabcdefgabcdabcdefgabcdefgabcdefgabcdefcdefg

EDKYEEEIKVLSDK---LKEAETRAEFAERSVTKLEKSIDDLEDE----LYAQKLKYKAISEEMKQLEDKVEELLSK----

abcdefgabcdefg---abcdefgabcdefgabcdefgabcdefg----abcdefgabcdefgabcdefgabcdefg

**Modeling method:**

The medial tail of Human myosin VI was modeled using MODELLER software [1]. The template used for modeling is 2EFR coiled-coil domain. Twenty models were generated and the best model was selected based on energy. The best model was minimized using SYBLY package (SYBYL7.1, Tripos Inc) to remove the short contacts; minimization was carried out till the energy of the system becomes negative. The quality of the minimized structure was checked using PROCHECK [2].

1. Sali A, Blundell TL. Comparative protein modelling by satisfaction of spatial restraints. *J Mol Biol*. 1993;234:779–815.

2. Laskowski RA, Mac Arthur MW, Moss DS, Thornton JM. PROCHECK: A program to check the stereo chemical quality of protein structures. *J Appl Crystallogr*. 1993;26:283–91.
